# Study on the Interaction between the Characteristics of Retinal Microangiopathy and Risk Factors for Cerebral Small Vessel Disease

**DOI:** 10.1155/2022/9505945

**Published:** 2022-06-09

**Authors:** Qian Tang, Yanli Zhang, Zhengfang Yang, Siou Li, Meini Wu, Yongming Guo, Weina Zhao, Changhao Yin

**Affiliations:** ^1^Department of Neurology, Hongqi Hospital Affiliated to Mudanjiang Medical University, Mudanjiang, China; ^2^Heilongjiang Key Laboratory of Ischemic Stroke Prevention and Treatment, Mudanjiang, China; ^3^Department of Neurology, The People's Hospital of Anyang City, Anyang, China; ^4^Department of Anesthesiology, Hongqi Hospital Affiliated to Mudanjiang Medical University, Mudanjiang, China; ^5^Department of Endocrinology, Hongqi Hospital Affiliated to Mudanjiang Medical University, Mudanjiang, China

## Abstract

**Objective:**

This study was designed to explore the characteristics of retinal microangiopathy in patients with cerebral small vessel disease (CSVD) and clarify its interaction with the risk factors for CSVD.

**Methods:**

Sixty patients with CSVD and 15 healthy individuals were enrolled. Demographic data, risk factors, and medical history were recorded, and magnetic resonance imaging was performed to detect and analyze the characteristics of retinal microangiopathy in the two groups. The interaction among retinal microangiopathy, vascular risk factors, and total imaging load of CSVD was compared.

**Results:**

(1) Hypertension, standard deviation of systolic blood pressure (SBPSD), standard deviation of blood glucose (SDBG), and atherogenic index of plasma (AIP) were independent vascular risk factors for CSVD. (2) Statistically significant differences in hypertension, SBPSD, SDBG, and AIP were observed between the two groups in terms of the total imaging burden of CSVD (*p* < 0.05). (3) Multivariate logistic linear regression showed that CSVD was associated with a wider central retinal vein equivalent (CRVE) (*p* = 0.015), a smaller arteriole-to-venule ratio (AVR) (*p* = 0.001), and a higher incidence of vessel tortuosity (*p* = 0.027). (4) When the total imaging burden of CSVD ranges from 0 to 4 points, the CRVE is larger, the AVR is smaller, and the incidence of vascular tortuosity is higher, with a statistically significant difference (*p* < 0.05). (5) The characteristics of retinal microangiopathy were correlated with hypertension, SBPSD, SDBG, and AIP (*p* < 0.05). (6) An association was observed between the characteristics of retinal microangiopathy and vascular risk factors and the total imaging burden of CSVD (*p* < 0.05).

**Conclusions:**

(1) Hypertension, SBP variability, BG fluctuation, and AIP are independent vascular risk factors for CSVD. (2) Retinal microvessels are changed in patients with CSVD, and venous dilatation, decreased arteriovenous ratio, and vascular tortuosity are the main characteristics of the disease. (3) The characteristics of retinal microangiopathy are interactively correlated with the total imaging load and risk factors for CSVD and can be used as indicators of the severity of CSVD.

## 1. Introduction

Cerebral small vessel disease (CSVD) refers to the pathological changes in cerebral microarteries, small arteries, capillaries, and small veins with several etiologies, including atherosclerotic disease, cerebral amyloid angiopathy, and immune-mediated, genetic, infectious, and radiation-induced causes, which is characterized by high incidence, insidiousness, and poor prognosis. However, the pathogenesis of this disease is unknown, which has recently gained so much attention in the field of stroke. Several risk factors related to stroke cause damage to small arteries and veins through a series of complex pathological processes, which can be observed in pathological or imaging examinations; these risk factors can lead to dementia in 45% of stroke cases [1–3]. Additionally, among patients with these risk factors, 20%–25% experience stroke, and subsequently, they can accelerate the progression of stroke [4]. Depending on the location, CSVD can cause several symptoms, such as mood disorders and motor and gait dysfunction. Current studies have shown that increasing age, hypertension, hyperlipidemia, diabetes, and smoking, among others, are independent risk factors for CSVD [5–7]. Based on imaging classification, CSVD includes lacunar infarcts (LIs), recent subcortical small infarcts (RSSIs), white matter hyperintensities (WMHs), enlarged perivascular space (EPVS), cerebral microbleeds (CMBs), and cerebral atrophy. CSVD has a complex etiology, causes insidious and diverse clinical symptoms, and relies mainly on imaging for diagnosis. The diagnosis of this disease is limited because of the lack of clinical diagnostic criteria combined with pathophysiological mechanisms, and CSVD is difficult to directly observe in the human body, so visual imaging techniques are used to visualize small arteries. One of the imaging techniques associated with CSVD is retinal vascularization [8], where advanced imaging modalities can detect signs of disease earlier than current standard imaging techniques. Early detection and positive intervention can have positive implications for improving patient prognosis.

The retinal microvasculature is an extension of cerebral blood vessels. The central retinal artery is a branch of the ophthalmic artery, which is a peripheral artery, homologous to the middle cerebral artery and the choroid plexus artery; both originate from the internal carotid artery and enter the fundus after branching out into small branches. The central retinal artery has the same embryological origin and vascular pathway as small cerebral blood vessels, with a diameter of approximately 50–250 *μ*m. Therefore, changes in the retinal microvasculature may provide a new perspective on the mechanism of CSVD. The process of microangiopathy is as follows: functional changes in the microcirculation, endothelial injury, basement membrane thickening, increase in blood viscosity, erythrocyte aggregation, platelet adhesion and aggregation, and finally microthrombosis or microvascular occlusion. Retinal vascular characteristics include retinal vascular imaging measures (i.e., retinal arteriovenous diameter and arteriovenous ratio), clinical retinal diseases (e.g., hypertensive retinopathy and diabetic retinopathy), macular degeneration, retinal microhemorrhage, retinal microaneurysms, retinal arteriovenous occlusion, and optic disk disease. Many studies have used different retinal imaging tools to examine the association between retinal microangiopathy and CSVD, including fundus photography and optical coherence tomography angiography [9, 10]. However, due to technical limitations, retinal microangiomas, retinal microhemorrhages, neovascularizations, and other retinal microangiopathy features could not be observed, and the findings were inconsistent. Retinal microvessels share common anatomical, histological, and pathophysiological features with small brain vessels and are the only directly observable microvessels on the human body. Vascular risk factors mainly damage the retina and brain in the form of vascular diseases, so we speculate that the alterations of retinal microvessels parallel the pathological changes in cerebral small vessels and speculate that the lesion parameters of retinal microvessels may directly reflect the severity of CSVD. In this study, we used fundus fluorescein angiography (FFA) to examine the relationship between the characteristics of retinal microvascular disease and the severity of CSVD. FFA breaks through the previous method of static observation of the fundus and dynamically observes the retinal blood circulation with high resolution and sensitivity. This study will provide a new perspective on the pathogenesis of CSVD by clarifying the interaction between the characteristics of retinal microvascular changes and the total imaging load and vascular risk factors for CSVD.

## 2. Materials and Methods

### 2.1. Subjects Collected

Data on patients diagnosed with CSVD in the outpatient or inpatient department of Hongqi Hospital affiliated to Mudanjiang Medical College between July 2019 and September 2020 were collected. Patients with CSVD must be diagnosed using magnetic resonance imaging (MRI) using a 3.0T MRI scanner in the last 3 months. Additionally, volunteers with normal brain MRI findings in the same age group in the same period were recruited at the physical examination center as the normal control (NC) group. After informed consent was obtained, a binocular FFA examination was performed, and 75 patients were finally included in the study (60 patients in the CSVD group and 15 subjects in the NC group). The CSVD group was selected as the study group. The CSVD group included 19 males (31.67%) and 41 females (68.33%). The age of the patients in the CSVD group ranged from 47 to 78 years, with an average of 61.85 ± 7.43 years. The NC group included six males (40.00%) and nine females (60.00%), with an average age of 58.27 ± 6.66 years (range, 48–69 years). This study was approved by the Ethics Committee of Mudanjiang Medical College, and all enrolled patients were informed about the study and signed the informed consent form. The diagnosis of CSVD was according to the 2015 Chinese Consensus on the Diagnosis and Treatment of Cerebral Small Vessel Disease [11] completion of brain MRI with any one or more of the following: RSSI, LI, lacuna, WMH, EPVS, CMBs, and cerebral atrophy, among others.

To be eligible for inclusion in the study, patients should meet the following inclusion criteria: (1) have been admitted within 48 h of onset and attended outpatient and emergency clinics, undergone MRI, and met the aforementioned diagnostic criteria for CSVD; (2) age ≥18; (3) have undergone binocular FFA examination and have been analyzed by the same physician; and (4) have agreed to participate or have family members that agreed to their participating in this study and have signed the informed consent form.

Patients were excluded from the study if they met any of the following exclusion criteria: (1) had FFA contraindications; (2) had hereditary CSVD (e.g., cerebral autosomal dominant arteriopathy with subcortical infarcts and leukoencephalopathy, cerebral autosomal recessive arteriopathy with subcortical infarcts and leukoencephalopathy, and Fabry disease); (3) had multiple sclerosis, neuromyelitis optica, and other autoimmune-related diseases; (4) had recent eye diseases or a history of fundus surgery; (5) had severe cataracts or who are unable to dilate due to mydriasis drugs, glaucoma, and anterior chamber angle stenosis, among others, which affect the observation of the fundus; (6) had severe cardiac failure, hepatic failure, renal failure, epilepsy, craniocerebral trauma, severe infection, malignant tumor, and history of asthma, among others; (7) had pregnancy; (8) had possible cardiogenic embolism, large atherosclerotic cerebral infarction, and other suspected non-CSVD neurological diseases; and (9) refused to participate or did not consent to participate in the experiment.

### 2.2. General Data Collection and Risk Factors for CSVD

For patients who met the inclusion and exclusion criteria, the following materials were collected: (1) general information including gender, age, and ethnicity; (2) personal history [12] including smoking and alcohol consumption; and (3) past history [13] including hypertension, diabetes, coronary atherosclerotic heart disease, atrial fibrillation, and valvular heart disease.

### 2.3. Laboratory Inspection

The subjects must fast, and after fasting, 5 ml of venous blood from the forearm was drawn on the day of the outpatient visit or the morning of hospitalization to examine blood routine and biochemical indicators. All blood samples were tested in the Laboratory of Hongqi Hospital affiliated to Mudanjiang Medical College, including fasting blood glucose, platelet count, creatinine, blood urea nitrogen, uric acid, total cholesterol (TC), triglyceride (TG), high-density lipoprotein cholesterol (HDL-C), and low-density lipoprotein cholesterol (LDL-C). The atherogenic index of plasma (AIP) value was calculated using the following formula: AIP = log (TG/HDL-C).

### 2.4. Systolic (SBP) and Diastolic Blood Pressure (DBP) and Blood Pressure Variability

The subjects were instructed to wear an ambulatory blood pressure monitor (model D1) on the upper arm to measure the brachial artery blood pressure. The daytime blood pressure was set from 6 am to 10 pm, and the measurement was performed every 30 min. The night blood pressure was set from 10 pm to 6 am, and the measurement was performed automatically every 1 h. The monitoring indicators included 24 h average SBP and 24 h average DBP. The blood pressure variability within 24 h was calculated according to the results of blood pressure monitoring. Blood pressure variability is mainly expressed by the standard deviation of systolic blood pressure (SBPSD) and the standard deviation of diastolic blood pressure (DBPSD).

### 2.5. Assessment of Blood Glucose Fluctuations

Using the self-monitoring method of blood glucose, the fish-leap blood glucose meter was used to monitor the fingertip blood glucose levels before and after three meals and before bedtime, and the times of the three meals and bedtime were 08:00, 12:00, 18:00, and 21:00, respectively. The premeal blood glucose level was measured 10 min before the meals and calculated from the first bite of food, and the postmeal blood glucose level was measured 2 h later. The fingertip blood glucose level was measured seven times in the entire process, and the standard deviation of blood glucose (SDBG) was calculated.

### 2.6. Imaging Evaluation of CSVD

**Figure 1:**
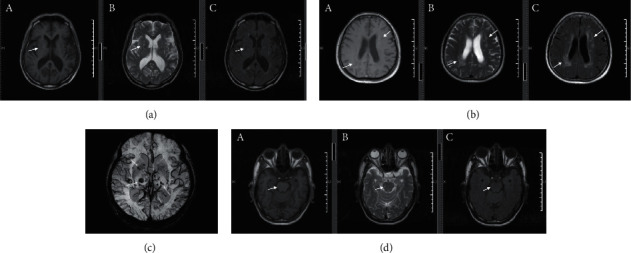
Different image types of CSVD. (a) The lesion showed low signal intensity on T1WI (A); the lesion showed high signal intensity on T2WI (B); the lesion showed low signal intensity on FLAIR (C). (b) Low signal in the anterior and posterior horns of the lateral ventricle on T1WI (A); high signal in the anterior and posterior horns of the lateral ventricle on T2WI (B); high signal in the anterior and posterior horns of the lateral ventricle on FLAIR (C). (c) Lesions showed a low signal on SWI. (d) The brainstem showed a low signal on T1WI (A); the brainstem showed a high signal on T2WI (B); the brainstem showed a high signal on FLAIR (C).

All brain MRI imaging and clinical data were blinded and interpreted independently by two experienced neurologists. The MRI markers were defined according to the CSVD imaging diagnostic criteria published in the STRIVE study [14]:

  LI: a round or oval lesion involving subcortical areas on MRI with a diameter of 3–15 mm, cerebrospinal fluid-filled cavity with a low signal on T1-weighted imaging (T1WI) and fluid-attenuated inversion recovery (FLAIR) (Figure 1(a)), and high signal on T2-weighted imaging (T2WI).
  WMH: abnormal signal around the lateral ventricles and deep white matter on MRI, with a slightly low or equal signal on T1WI and high signal on T2WI and FLAIR (Figure 1(b)), with relatively symmetrical bilateral lesions.
  CMBs: not easily observed on T1WI and T2WI sequences, but round or ovoid low signal lesions with well-defined borders on T2-weighted gradient-echo or susceptibility-weighted imaging (SWI) sequences (Figure 1(c)).
  EPVS: clear boundary on MRI; round, oval, tubular, or linear, with consistent alignment with penetrating vessels, the same signal as cerebrospinal fluid, low signal on T1WI (Figure 1(d)) and FLAIR; and high signal on T2WI. No occupancy or contrast enhancement effect.

The aforementioned CSVD imaging markers constitute the total baseline CSVD imaging load. The sum of LI, WMH, CMBs, and EPVS in MRI manifestations ranges from 0 to 4 points, which represents the total CSVD imaging load, and the higher the score, the more severe the CSVD damage to brain tissue [15]. The total imaging load of CSVD includes the following: number of LI ≥ 1, number of CMBs ≥1, moderate to severe (>10) basal ganglia EPVS, and WMH periventricular or deep Fazekas score ≥3. The sum of the aforementioned parameters is the total CSVD imaging load (0–4 points).

### 2.7. FFA Examination

All FFA images were collected at the Ophthalmology Department of Hongqi Hospital affiliated to Mudanjiang Medical College, using a German Heidelberg fundus contrast machine (model HRAplusII). The FFA examination was performed by the same ophthalmologist on the same day as the outpatient clinic visit or the next day of the inpatient treatment after assessing the patient's general status. Atropine was used to dilate both eyes 30 min before the surgery, and 0.1 mL of 20% sodium fluorescein was added to 10 mL of 0.9% sodium chloride injection solution and mixed thoroughly. Then, the mixture was slowly injected into a forearm vein to test for allergies. The injection site was observed for 5 min; no adverse reactions indicated that the sodium fluorescein allergy test was negative. Then, 3 mL of 20% sodium fluorescein solution was rapidly pushed to the elbow vein within 4-5 s, and after 5 s, both of the patient's eyes were photographed continuously. After the veins were filled, photographs were taken intermittently for 15 min, and the patient was closely monitored for discomfort during the process. After the angiography, the patient must stay for 10 min and leave if there were no adverse reactions. The FFA images were uploaded to a computer, and an FFA report was issued by a professional ophthalmologist, which contains the following information: retinal arteriovenous diameter, vessel shape and morphology, neovascularization, retinal hemorrhage, retinal microangioma, optic disk edema, optic disk hemorrhage, fluorescence leakage, macular degeneration, hypertensive retinopathy, and diabetic retinopathy, among others. These pieces of information were used to summarize the characteristics of retinopathy. Six large retinal arteries and veins with optic disk diameters within the range of 0.5–1.0 from the edge of the optic disk were selected, and ImageJ was used to quantitatively analyze each frame of the FFA images; then, the central retinal artery equivalent (CRAE), central retinal vein equivalent (CRVE), and arteriole-to-venule (AVR) ratio were calculated. The AVR ratio is the ratio of the CRAE to the CRVE. The normal AVR ratio is 2:3. Retinal artery stenosis and/or venous dilation can decrease the AVR ratio.

### 2.8. Statistical Analysis

Statistical Package for the Social Sciences, version 25.0, was used to perform all statistical analyses. The Kolmogorov–Smirnov test was used to verify whether each group of continuous variables conformed to a normal distribution. The measurement data of the normal distribution are represented by means ± standard deviations (*x* ± *s*), and the count data are represented by frequencies (%). The *t*-test was used for independent sample comparison between two groups, and an analysis of variance was used for comparing multiple groups. Then, in the univariate analysis of count data, the chi-square test was used, and the test level was *α* = 0.05; a logistic regression analysis was used for the multivariate analysis. The Spearman rank correlation test was used to determine the correlation between the total imaging load of CSVD and the vascular risk factors and the characteristics of retinal vascular disease. A multiple linear regression model was developed to analyze the linear relationship between retinal microvascular disease and vascular risk factors. Multivariate variance analysis was used to analyze the interaction between retinal microvascular disease, vascular risk factors, and the total imaging load of CSVD. A paired *t*-test was used to calculate the retinal arteriovenous diameter in both eyes; no statistical difference in the diameters of bilateral retinal arteries and veins was observed. The average value of the bilateral arteriovenous diameters was calculated for statistical analysis.

## 3. Results

### 3.1. Baseline Characteristics

We recruited 60 patients with CSVD (CSVD group) and 15 health individuals (NC group). The gender and ethnicity of the two groups of subjects were assessed using the chi-square test, and age was assessed using a one-way analysis of variance. The results indicated that age, gender, and ethnicity were not significantly different between the two groups (*p* > 0.05). The comparison of the general demographic data of the subjects is shown in Table 1.

### 3.2. Comparison of the Risk Factors for CSVD

We compared vascular risk factors between the CSVD and NC groups (Supplemental Table S1), and significant differences (*p* < 0.05) in hypertension (*p* = 0.012), diabetes (*p* = 0.005), coronary heart disease (*p* = 0.038), 24-h average SBP (*p* = 0.004), SBPSD (*p* = 0.010), SDBG (*p* = 0.021), and AIP (*p* = 0.037) were found; however, no significant differences in smoking history, drinking history, 24 h average DBP, DBPSD, fasting blood glucose, TG, TC, HDL-C, LDL-C, urea, creatinine, uric acid, and platelet count were observed between the two groups (*p* > 0.05).

Logistic regression analysis models were developed with the presence or absence of CSVD as the dependent variable and hypertension, diabetes mellitus, coronary heart disease, 24-h mean SBP, SBPSD, SDBG, and AIP as independent variables. The results showed that hypertension, SBPSD, SDBG, and AIP were independent risk factors for the development of CSVD (*p* < 0.05) (Table 2).

### 3.3. Correlation between Independent Vascular Risk Factors and the Total Imaging Load of CSVD

**Figure 2:**
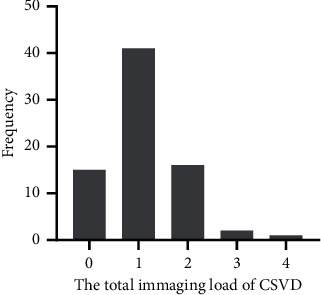
Distribution of the total imaging load of CSVD in terms of the number of people.

**Figure 3:**
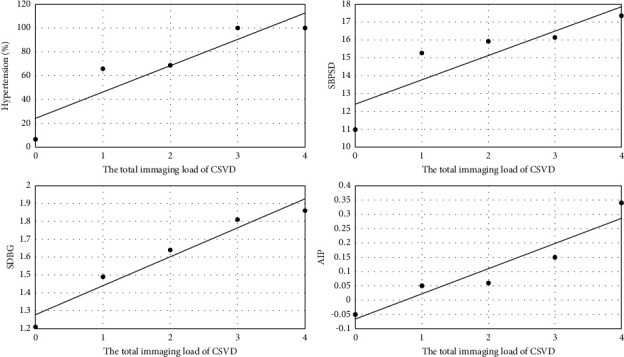
Hypertension, SBPSD, SDBG, and AIP and the total imaging load of CSVD.

Regarding the total imaging load of CSVD, 15 cases had 0 points, 41 cases had 1 point, 16 cases had 2 points, two cases had 3 points, and one case had 4 points (Figure 2). Hypertension (*p* = 0.002), SBPSD (*p* = 0.021), SDBG (*p* = 0.018), and AIP (*p* = 0.040) were significantly different among the CSVD imaging load groups (*p* < 0.05). The results are shown in Table 3 and Figure 3.

### 3.4. Analysis of the Characteristics of Retinal Microvascular Disease

**Figure 4:**
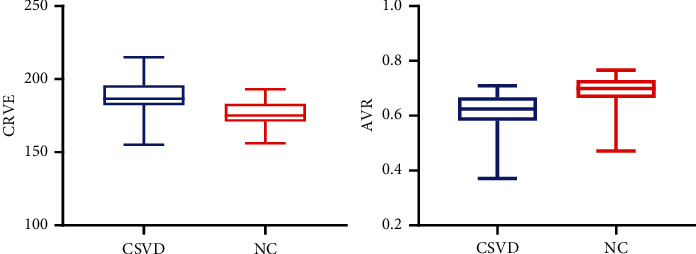
Comparison of CRVE and AVR between the CSVD and NC groups.

**Figure 5:**
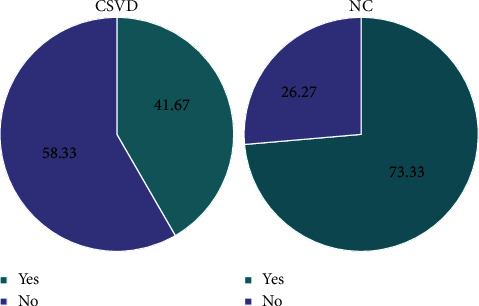
The proportion of retinal vascular tortuosity in the CSVD and NC groups.

**Figure 6:**
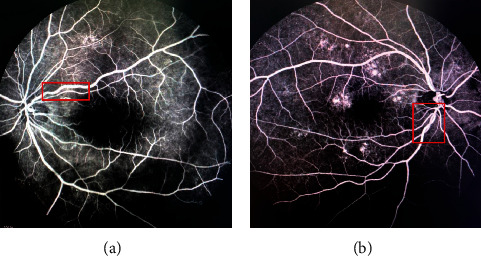
Features of retinal microvascular disease. (a) CRVE increases; AVR decreases. (b) Tortuous retinal blood vessels.

Statistically significant differences in CRVE, AVR, and vascular tortuosity were observed between the two groups (*p* < 0.05). However, no significant differences in CRAE, neovascularization, retinal hemorrhage, retinal hemangioma, omental circulation time, optic disk hemorrhage, optic disk edema, optic disk fluorescence leakage, macular edema, diabetic retinopathy, and hypertensive retinopathy were found between the two groups (*p* > 0.05). The results are shown in Table 4. The comparison of CRVE and AVR between the two groups is shown in Figure 4, and the comparison of vascular tortuosity is shown in Figure 5. The CRVE increased, and the AVR decreased in the FFA images shown in Figure 6(a), and the FFA image of retinal vascular tortuosity is shown in Figure 6(b).

### 3.5. Correlation between Retinal Microvascular Disease and the Total Imaging Load of CSVD

**Figure 7:**
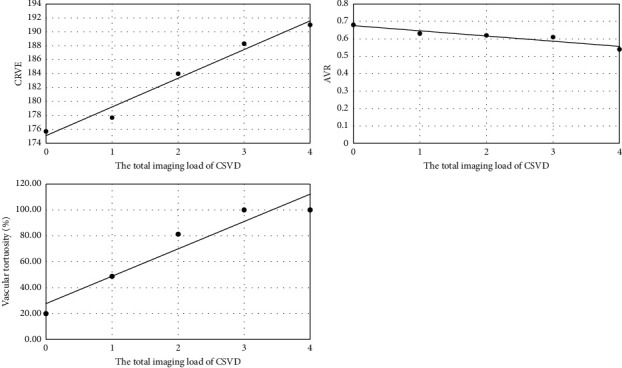
CRVE, AVR, vascular tortuosity, and the total imaging load of CSVD.

In the different CSVD imaging total load groups, CRVE became increasingly larger, with a statistically significant difference (*p* = 0.009), AVR became significantly smaller and negatively correlated with the total imaging load of CSVD (*p* = 0.001), and the incidence of vascular tortuosity became increasingly higher, with a statistically significant difference (*p* = 0.014). The results are shown in Table 5 and Figure 7.

### 3.6. Correlation between Retinal Microvascular Disease and Vascular Risk Factors

Multivariate linear regression analysis was performed using retinal microvascular disease as the dependent variable and hypertension, SBPSD, SDBG, and AIP as the independent variables. The results showed that after stepwise regression analysis, the characteristics of the retinal microvascular disease were correlated with hypertension, SBPSD, SDBG, and AIP (*p* < 0.05). The results are shown in Table 6.

### 3.7. Interaction between the Characteristics of Retinal Microvascular Disease, Vascular Risk Factors, and the Total Imaging Load of CSVD

The comparison of the characteristics of retinal microvascular disease with the total imaging load of CSVD and independent vascular risk factors (*p* < 0.05) suggested an interaction between the characteristics of retinal microvascular disease, vascular risk factors, and the total imaging load of CSVD. The results are shown in Table 7.

## 4. Discussion

This study included the total imaging load of CSVD and clarified the interaction between the features of retinal microvascular change, the total imaging load of CSVD, and vascular risk factors. The characteristics of the retinal microvascular disease may provide theoretical evidence for the pathogenesis of CSVD.

### 4.1. Analysis of Vascular Risk Factors for Cerebral Small Vessel Disease

#### 4.1.1. Blood Pressure and CSVD

This study [16] found that hypertension is an independent risk factor for CSVD, which is consistent with the results of previous studies. The mechanism is that long-term hypertension leads to hyaline degeneration of lipids in the media, stenosis of small arteries and small perforating arteries, thickening of the vessel wall, increased vascular fibrosis that results in vascular wall hardening and reduction of cerebral blood flow, and secondary blood–brain barrier damage, oxidative stress, neuroinflammation, and endothelial dysfunction, among others, which lead to brain hypoxia and impaired neurological function. CSVD is an important cause of hypertension-induced cognitive impairment and one of the most common causes of vascular dementia [17, 18]. The standard deviation of blood pressure is often used to evaluate blood pressure variability in clinical practice. Blood pressure variability is related to vascular smooth muscle dysfunction and vascular endothelial dysfunction [19]. Compared with the average blood pressure, blood pressure variability can better predict the occurrence of cerebrovascular events. Several studies support this theory [20, 21]. van Middelaar et al. [22] showed in a meta-analysis of four studies on the effect of hypertensive drugs on CSVD that patients in the intensive hypertensive drug treatment group had significantly less WMH progression, suggesting that hypertensive drugs have a protective effect on the progression of WMHs.

#### 4.1.2. Blood Glucose and CSVD

The SDBG index was analyzed in this study, reflecting the degree of blood glucose fluctuations. Studies have found that SDBG is an independent risk factor for CSVD. High blood glucose levels and disrupted insulin regulation in patients with diabetes lead to vascular endothelial damage and oxidative stress in the body, which damage blood vessels and neurons and lead to the formation of atherosclerosis. Studies have shown that blood glucose fluctuation is an important vascular risk factor independent of diabetes mellitus, representing excessive blood glucose excursions that induce apoptosis, metabolic disorders, and inflammatory responses by triggering oxidative stress and affecting cytokine production [23].

#### 4.1.3. AIP and CSVD

Abnormal blood lipid metabolism can lead to endothelial lipid deposition and accelerate the development of vascular atherosclerosis. TG and HDL-C are the most common blood lipid indicators. Increased TG levels can cause vascular disease, and HDL-C is a protective factor for the vasculature and is associated with a lower risk of CSVD and lower WMH volume [24]. Dobiasova et al. described AIP as a biomarker of plasma atherosclerosis for the first time. AIP is often used to assess the degree of atherosclerosis and to predict the severity of lipid metabolism disorders beyond individual lipids and/or TC/HDL-C ratios. Several studies have confirmed the association of AIP with metabolic syndrome, carotid plaque, and renal insufficiency, among others [25, 26]. Additionally, recent studies have shown that AIP is a strong marker for predicting the risk of atherosclerosis and cardiovascular disease, and cardiovascular disease risk factors are related to the integrity of brain structures and cognitive impairment [27]. Studies on the application of AIP to CSVD remain limited. This study demonstrated that AIP is an independent vascular risk factor, which may be a sensitive index that can be used to study CSVD in the future.

### 4.2. Correlation Analysis of Vascular Risk Factors and the Total Imaging Load of CSVD

The total imaging load of CSVD is based on the number and type of each CSVD imaging marker displayed on MRI, the corresponding value is assigned, and the cumulative sum is the total imaging load of CSVD, which can reflect the severity of CSVD, thus estimating the full impact of CSVD on the brain more accurately in a simple and practical way, and this index has a more practical value than a single imaging marker [28]. The results of a study using home self-measured blood pressure showed that blood pressure variability was associated with the total imaging load of CSVD, but not with diastolic variability [29]. Another study has found that 24 h day and night SBP levels and SBP variability were positively correlated with the total imaging load of CSVD [20]. There are few related studies on the total imaging load of CSVD and vascular risk factors, and the correlation between vascular risk factors and CSVD severity can be further investigated by expanding the sample size.

### 4.3. Characteristics of Retinal Microvascular Disease in Patients with CSVD

The relationship between retinal microvascular disease and CSVD has received increasing attention [30–32], and the most commonly used metrics related to measuring retinal microvascular diameter are CRAE, CRVE, and AVR. In this study, we found that patients with CSVD had a wider CRVE and lower AVR. Several related studies have been conducted in the past; however, the results are different and there is no uniform conclusion. A study [33] has shown that retinal microangiopathy is associated with extracranial stenosis of the internal carotid artery, with a wider CRVE and lower AVR. A Rotterdam study proved for the first time that the relationship between retinal vein dilatation and the occurrence and development of CSVD is significantly higher than that of retinal artery stenosis, while the retinal vessel diameter has no significant correlation with the severity of white matter lesions and the incidence of LI [34]. Yatsuya et al. [35] found a positive correlation between narrowing of the central retinal artery diameter and widening of the central retinal vein diameter and LI. However, Ji et al. [36] found that a narrowing of the central retinal artery diameter and a widening of the central retinal vein diameter were associated with deep white matter lesions. Mutlu et al. [37] showed by subgroup analysis of the Rotterdam study that narrowing of the central retinal artery diameter and widening of the central retinal vein diameter were correlated with EPVS in hemianopia, but not with EPVS in the basal nucleus. A study pooling the results of the Beaver Dam Eye Study and the Blue Mountains Eye Study showed that smaller arterioles and larger venules in the retina were associated with an increased risk of stroke-related death [38]. Dumitrascu et al. [39] found that both small venous dilatation and small artery stenosis were associated with LI, with small artery stenosis remaining associated with cerebral WMH. The CHS study [40] found that fundus venous dilatation was positively associated with an increased incidence of stroke over the next 5 years and that the relative risk of ischemic stroke increased as the AVR decreased. Overall, there are few large-scale and prospective studies on the relationship between CSVD and the characteristics of retinal microvascular disease. This study did not find a correlation between the central retinal artery and CSVD, considering the possible reason that retinal microangiopathy is caused by retinal hypoxia, and the main components of the vessel wall are endothelial cells and pericytes, which are very sensitive to ischemia and hypoxia. The retina can increase the production of nitric oxide and reactive oxygen species in an ischemic and hypoxic environment, which activates the associated inflammatory factors. Retinal microvascular venous dilatation is a manifestation of retinal ischemia and hypoxia, and venous stasis can lead to hypoperfusion and impaired removal of cellular metabolic waste products, thereby exacerbating retinal microangiopathy. Because of a series of vascular risk factors that lead to retinal ischemia and hypoxia, venous dilatation is a compensatory effect to increase the blood supply to the retina. The retinal vessel diameter is independently associated with end-organ damage, and studies have shown that retinal vein dilation increases the risk of ischemic stroke [41]. The CRVE and AVR, as sensitive and easily available biomarkers, add value for predicting the risk of cerebrovascular disease.

This study found that vascular tortuosity is the main feature of retinal vasculopathy in CSVD. The normal central retinal vein was distributed parallel to the central retinal artery, and the two traveled together. Local or systemic pathological changes can lead to tortuous expansion of the retinal microvasculature in a spiral or “wall-like” pattern. There are several possible pathogenic mechanisms for retinal microvascular tortuosity: (1) hemodynamic factors: diseases of various systems throughout the body can lead to changes in blood oxygen content and vascular endothelial damage, among others, which can cause hemodynamic changes. After blood flow increases, the body may have adaptive changes, such as venous vasodilation; however, arterial vessels cannot expand rapidly, resulting in vascular tortuosity; (2) myotonic factors: Brancher [42] believes that metabolites (e.g., lactic acid), blood gas, and mediators (e.g., prostaglandins) can alter the tone of vascular smooth muscles, which are normally arranged in a spiral layer and are relaxed in hypoxia, leading to an increase in width and length, thus causing vascular tortuosity; (3) vascular endothelial growth factors (VEGFs): VEGFs help maintain the stability of the vascular structure. Under pathological conditions, a large number of VEGFs are distributed in the retina. VEGFs increase endothelial cell pinocytosis vesicle trafficking and induce the production of phosphorylated proteins. The phosphorylated proteins produced can be ubiquitinated, which reduces the stability of tight junctions and destroys the role of the blood–retina barrier. The central retinal vein lacks a pericyte sheath and therefore may exhibit an enlarged CRVE, while the central retinal artery is surrounded by a pericyte sheath, resulting in an inability to rapidly increase the vessel diameter, resulting in a tortuous vessel. We believe that the vascular tortuosity observed in this study is related to systemic multisystemic factors leading to retinal ischemia and hypoxia, and the mechanism of its occurrence may be a combination of multiple factors: a retinal microvascular disease associated with hypertension, systolic pressure variation coefficient, and AIP, which can cause a decrease in blood oxygen content or a compensatory increase in blood flow, and the occurrence of vascular smooth muscle relaxation, which triggers vascular tortuosity.

### 4.4. Correlation Analysis of the Total Imaging Load of CSVD and Retinal Microangiopathy

This study found that CRVE was positively correlated with the total imaging load of CSVD. AVR was negatively correlated with the total imaging load of CSVD. In the total imaging load of CSVD of 0–4 points, the diameter of the central retinal vein is getting wider and wider, with a significantly smaller AVR and an increasing incidence of vascular tortuosity.

### 4.5. The Interaction between the Characteristics of Retinal Microvascular Disease and the Total Imaging Load of CSVD and Vascular Risk Factors

This study found that hypertension, SBPSD, SDBG, and AIP were independent vascular risk factors for retinal microangiopathy in CSVD. The characteristics of retinal microvascular lesions (i.e., CRVE, AVR, and vascular tortuosity) were strongly correlated with the total imaging load of CSVD. There is an interaction between the characteristics of retinal microvascular lesions and the total imaging load of CSVD and independent vascular risk factors so that patients with CSVD with hypertension, large blood pressure variability, large fluctuations in blood glucose, and dyslipidemia were more likely to show the following retinal imaging findings: venous dilatation, decreasing AVR, and vascular tortuosity, among others. The characteristics of retinopathy are more significant and are closely related to the severity of CSVD.

Vascular risk factors are independent and important risk factors for CSVD and retinal microvascular disease. CSVD and retinal microangiopathy are the results of the combined effects of hypertension, diabetes, and other vascular risk factors through vascular damage pathways, such as reduced blood flow, destroying the blood–brain barrier and the integrity of the blood–retina barrier, among others. Vascular risk factors are closely related to the occurrence and development of CSVD and retinal microangiopathy, both of which are pathologically based on small vessel lesions, and the pathological changes of both are parallel. Patients with CSVD are prone to develop retinal microangiopathy at the same time due to reduced small vessel blood flow and impaired barrier function.

This study clarified that independent vascular risk factors were related to retinal microvascular disease, that is, CRVE, AVR, and vascular tortuosity parameters as retinal microangiopathy characteristics responded to the extent of vascular damage caused by vascular risk factors. Moreover, independent vascular risk factors were correlated with the total imaging load of CSVD, reflecting the severity of CSVD. It is speculated that retinal microvascular parameters can directly reflect the total imaging load of CSVD; that is, retinal microangiopathy characteristic parameters can be used as indicators of CSVD severity, and FFA can provide some clinical basis for CSVD. Therefore, a wider CRVE, lower AVR, and vascular tortuosity can be used as early indicators of retinal microvascular disease, reflecting the degree of vascular damage caused by vascular risk factors. The characteristics of the retinal microvascular disease are interactively related to the total imaging load of CSVD and vascular risk factors, which provide us with evidence-based medicine and help us better understand the predictive effect of retinal microvascular disease on the changes in CSVD. In our daily life, we can control vascular risk factors using oral antihypertensive drugs, lipid-lowering drugs, and hypoglycemic drugs, thereby reducing the risk of CSVD and retinal microvascular disease.

## Figures and Tables

**Table 1:** Comparison of the general demographic characteristics between the two groups.

Variables	CSVD group (*n* = 60)	NC group (*n* = 15)	*p* value
Age (years)	61.85 ± 7.43	58.27 ± 6.66	0.238
Male, *n* (%)	19 (31.67%)	6 (40.00%)	0.540
Ethnicity, Han Chinese (%)	56 (93.33%)	14 (93.33%)	0.178

*p* > 0.05 for age, gender, and ethnicity between the two groups. CSVD, cerebral small vessel disease; NC, normal control.

**Table 2:** Logistic regression analysis of different risk factors for CSVD.

Variables	Regression coefficient	Standard error	Wald	OR	*p* value
Hypertension	1.26	0.58	4.68	3.53	0.03^*∗*^
SBPSD	2.01	0.46	8.12	1.30	0.012^*∗*^
SDBG	0.24	0.67	2.31	1.06	0.017^*∗*^
AIP	1.72	0.47	5.72	3.21	0.025^*∗*^

^*∗*^The CSVD group compared with the NC group; *p* < 0.05. CSVD, cerebral small vessel disease; SBPSD, standard deviation of systolic blood pressure; SDBG, standard deviation of blood glucose; AIP, atherogenic index of plasma.

**Table 3:** Correlation between independent risk factors for CSVD and total imaging load of CSVD.

Variables	Total imaging load of CSVD					*p* value
	0 points (*n* = 15)	1 point (*n* = 41)	2 points (*n* = 16)	3 points (*n* = 2)	4 points (*n* = 1)	
Hypertension, *n* (%)	1 (6.67%)	27 (65.85%)	11 (68.75%)	2 (100.00%)	1 (100.00%)	0.002^*∗*^
SBPSD	10.99 ± 2.10	15.27 ± 2.10	15.92 ± 4.20	16.14 ± 3.10	17.35 ± 4.20	0.021^*∗*^
SDBG	1.21 ± 0.24	1.49 ± 0.29	1.64 ± 0.21	1.81 ± 0.32	1.86 ± 0.41	0.018^*∗*^
AIP	−0.05 ± 0.27	0.05 ± 0.30	0.06 ± 0.24	0.15 ± 0.07	0.34 ± 0.12	0.040^*∗*^

^*∗*^The independent risk factors for CSVD were correlated with the total CSVD imaging load; *p* < 0.05. CSVD, cerebral small vessel disease; SBPSD, standard deviation of systolic blood pressure; SDBG, standard deviation of blood glucose; AIP, atherogenic index of plasma.

**Table 4:** Comparison of the results of retinal microvascular parameters in the two groups of subjects.

Variables	CSVD group (*n* = 60)	NC group (*n* = 15)	*p* value
CRAE (*μ*m)	116.12 ± 10.99	119.73 ± 12.48	0.271
CRVE (*μ*m)	188.68 ± 10.73	175.73 ± 9.69	0.015^*∗*^
AVR	0.61 ± 0.05	0.68 ± 0.07	0.001^*∗*^
Neovascularization, *n* (%)	4 (6.67%)	1 (6.67%)	0.905
Vascular tortuosity, *n* (%)	35 (58.33%)	4 (26.67%)	0.028^*∗*^
Retinal hemorrhage, *n* (%)	13 (21.67%)	2 (13.33)	0.423
Retinal hemangioma, *n* (%)	13 (21.67%)	1 (6.67%)	0.483
Prolonged retinal circulation time, *n* (%)	13 (21.67%)	3 (20.00%)	0.724
Optic disk hemorrhage, *n* (%)	6 (10.00%)	1 (6.67%)	0.985
Optic disk edema, *n* (%)	3 (5.00%)	0	0.995
Optic disk fluorescence leakage, *n* (%)	30 (60.00%)	9 (60.00%)	0.108
Macular edema, *n* (%)	5 (8.33%)	3 (20.00%)	0.998

^*∗*^The CSVD group compared with the NC group; *p* < 0.05. CSVD, cerebral small vessel disease; NC, normal control; CRAE, central retinal artery equivalent; CRVE, central retinal vein equivalent; AVR, arteriole-to-venule ratio.

**Table 5:** Correlation of the total imaging load of CSVD with retinal microangiopathy.

Variables	Total imaging load of CSVD					*p* value
	0 points (*n* = 15)	1 point (*n* = 41)	2 points (*n* = 16)	3 points (*n* = 2)	4 points (*n* = 1)	
CRVE (*μ*m)	175.73 ± 9.69	177.69 ± 9.63	184.00 ± 12.54	188.29 ± 19.80	191.00	0.009^*∗*^
AVR	0.68 ± 0.07	0.63 ± 0.06	0.62 ± 0.03	0.61 ± 0.09	0.54	0.001^*∗*^
Vascular tortuosity, *n* (%)	3 (20.00%)	20 (48.78%)	13 (81.25%)	2 (100.00%)	1 (100.00%)	0.014^*∗*^

^*∗*^The total imaging load of CSVD was correlated with retinal microangiopathy; *p* < 0.05. CSVD, cerebral small vessel disease; CRVE, central retinal vein equivalent; AVR, arteriole-to-venule ratio.

**Table 6:** Comparison of correlation between retinal microvascular changes and vascular risk factors.

Variables	High blood pressure	SBPSD	SDBG	AIP
CRVE	0.022^*∗*^	0.037^*∗*^	0.024^*∗*^	0.015^*∗*^
AVR	0.025^*∗*^	0.026^*∗*^	0.015^*∗*^	0.029^*∗*^
Tortuous blood vessels	0.028^*∗*^	0.032^*∗*^	0.317	0.015^*∗*^

^*∗*^Retinal microvascular changes compared with vascular risk factors; *p* < 0.05. CSVD, cerebral small vessel disease; CRVE, central retinal vein equivalent; AVR, arteriole-to-venule ratio.

**Table 7:** The interaction between the characteristics of retinal microvascular disease, vascular risk factors, and the total imaging load of CSVD.

Total imaging load of CSVD	Vascular risk factors	CRVE	AVR	Tortuous blood vessels (%)
0	Hypertension	170.73 ± 9.84	0.68 ± 0.07	42.85
	SBPSD	175.72 ± 9.39	0.68 ± 0.03	28.57
	SDBG	177.73 ± 10.82	0.68 ± 0.05	26.67
	AIP	174.65 ± 7.36	0.67 ± 0.04	6.67

1	Hypertension	175.00 ± 9.64	0.63 ± 0.06	71.42
	SBPSD	176.69 ± 9.36	0.63 ± 0.05	42.86
	SDBG	177.89 ± 9.54	0.64 ± 0.01	65.85
	AIP	176.72 ± 7.65	0.63 ± 0.07	60.00

2	Hypertension	185.03 ± 9.84	0.62 ± 0.03	81.25
	SBPSD	186.00 ± 8.54	0.63 ± 0.01	88.89
	SDBG	181.09 ± 9.55	0.62 ± 0.05	83.33
	AIP	183.63 ± 11.24	0.62 ± 0.03	80.00

3	Hypertension	188.33 ± 9.85	0.61 ± 0.09	100.00
	SBPSD	188.33 ± 9.85	0.61 ± 0.09	100.00
	SDBG	188.29 ± 19.80	0.61 ± 0.09	50.00
	AIP	188.33 ± 9.85	0.61 ± 0.09	50.00

4	Hypertension	191.00	0.54	100.00
	SBPSD	191.00	0.54	100.00
	SDBG	191.00	0.54	100.00
	AIP	191.00	0.54	100.00

*F* value		1.204	0.664	0.278
*p* value		0.009^*∗*^	0.001^*∗*^	0.014^*∗*^

^*∗*^There was an interaction between the characteristics of retinal microangiopathy, vascular risk factors, and the total imaging load of CSVD; *p* < 0.05. CSVD, cerebral small vessel disease; CRVE, central retinal vein equivalent; AVR, arteriole-to-venule ratio; SBPSD, standard deviation of systolic blood pressure; SDBG, standard deviation of blood glucose; AIP, atherogenic index of plasma.

## Data Availability

The data used to support the findings of this study are available from the corresponding author upon request.

## Supplementary Materials

Supplementary Materials Table S1: comparison of the risk factors between CSVD and NC group.
