# Supplementary material for: Study on the Interaction between the Characteristics of Retinal Microangiopathy and Risk Factors for Cerebral Small Vessel Disease
Source: Contrast Media Mol Imaging. 2022 Jun 9;2022:9505945. doi: 10.1155/2022/9505945 (PMC9203197; doi:10.1155/2022/9505945)
Supplement: Supplementary Materials — Table S1: comparison of the risk factors between CSVD and NC group. [file 9505945.f1.doc]

| Table S1. Comparison of the risk factors between CSVD and NC group. | | | |
| --- | --- | --- | --- |
| Variables | CSVD group  (n = 60) | NC group  (n = 15) | *P-*value |
| Smoking history, n (%) | 21 (35.00%) | 2 (13.33%) | 0.128 |
| History of alcohol consumption, n (%) | 22 (36.67%) | 6 (40.00%) | 0.811 |
| Past History |  |  |  |
| Hypertension, n (%) | 41 (68.33%) | 1 (6.67%) | 0.012 ^*^ |
| Diabetes, n (%) | 32 (53.33%) | 2 (13.33%) | 0.005 ^*^ |
| CHD, n (%) | 14 (23.33%) | 0 | 0.038 ^*^ |
| Baseline measurements |  |  |  |
| 24-hour average SBP (mmHg) | 146.87 ± 20.41 | 129.00 ± 11.77 | 0.004 ^*^ |
| 24-hour average DBP (mmHg) | 83.00 ± 12.74 | 82.53 ± 13.88 | 0.901 |
| SBPSD | 16.35 ± 3.20 | 10.99 ± 2.10 | 0.010 ^*^ |
| DBPSD | 11.27 ± 3.52 | 12.32 ± 2.98 | 0.064 |
| Fasting blood glucose (mmol/L) | 7.13 ± 2.87 | 5.45 ± 0.83 | 0.054 |
| SDBG | 1.79 ± 0.31 | 1.21 ± 0.24 | 0.021 ^*^ |
| Platelets (10^9/L) | 217.97 ± 53.88 | 218.67 ± 51.57 | 0.964 |
| TC (mmol/L) | 5.10 ± 1.05 | 5.1007 ± 1.35 | 0.992 |
| TG (mmol/L) | 1.63 ± 0.99 | 1.34 ± 0.62 | 0.293 |
| HDL-C (mmol/L) | 1.34 ± 0.28 | 1.40 ± 0.34 | 0.497 |
| LDL-C (mmol/L) | 2.85 ± 0.73 | 3.03 ± 1.06 | 0.451 |
| AIP | 0.03 ± 0.26 | −0.05 ± 0.27 | 0.037 ^*^ |
| Urea (mmol/L) | 5.44 ± 1.32 | 5.05 ± 1.38 | 0.311 |
| Creatinine (μmol/L) | 62.49 ± 12.88 | 63.83 ± 15.53 | 0.729 |
| Uric acid (μmol/L) | 302.40 ± 93.13 | 314.13 ± 83.20 | 0.658 |
| *CSVD group compared with NC group, *p* < 0.05. CSVD, cerebral small vessel disease; NC, normal control; CHD, coronary atherosclerotic heart disease; SBP, systolic blood pressure; DBP, diastolic blood pressure; SBPSD, standard deviation of systolic blood pressure; DBPSD, standard deviation of diastolic blood pressure; SDBG, standard deviation of blood glucose; TC, total cholesterol; TG, triglyceride; HDL-C, high-density lipoprotein cholesterol; LDC-C, low-density lipoprotein cholesterol; AIP, atherogenic index of plasma | | | |
